# Rapid Manufacturing of Multilayered Microfluidic Devices for Organ on a Chip Applications

**DOI:** 10.3390/s21041382

**Published:** 2021-02-16

**Authors:** Roberto Paoli, Davide Di Giuseppe, Maider Badiola-Mateos, Eugenio Martinelli, Maria Jose Lopez-Martinez, Josep Samitier

**Affiliations:** 1 Nanobioengineering Group, Institute for Bioengineering of Catalonia (IBEC), Barcelona Institute of Science and Technology (BIST), 12 Baldiri Reixac 15–21, 08028 Barcelona, Spain; rob.paoli@gmail.com (R.P.); mbadiola@ibecbarcelona.eu (M.B.-M.); 2 Centro de Investigación Biomédica en Red en Bioingeniería, Biomateriales y Nanomedicina (CIBER-BBN), Monforte de Lemos 3–5, Pabellón 11, 28029 Madrid, Spain; 3 Department of Electronics and Biomedical Engineering, University of Barcelona, Martí i Franquès 1, 08028 Barcelona, Spain; 4 Department of Electronic Engineering, University of Rome “Tor Vergata”, 00133 Rome, Italy; di.giuseppe@ing.uniroma2.it (D.D.G.); martinelli@ing.uniroma2.it (E.M.); 5 Interdisciplinary Center for Advanced Studies on Lab-on-Chip and Organ-on-Chip Applications (IC-LOC), University of Rome Tor Vergata, 00133 Rome, Italy

**Keywords:** digital manufacturing, rapid prototyping, organ on a chip, microfluidics

## Abstract

Microfabrication and Polydimethylsiloxane (PDMS) soft-lithography techniques became popular for microfluidic prototyping at the lab, but even after protocol optimization, fabrication is yet a long, laborious process and partly user-dependent. Furthermore, the time and money required for the master fabrication process, necessary at any design upgrade, is still elevated. Digital Manufacturing (DM) and Rapid-Prototyping (RP) for microfluidics applications arise as a solution to this and other limitations of photo and soft-lithography fabrication techniques. Particularly for this paper, we will focus on the use of subtractive DM techniques for Organ-on-a-Chip (OoC) applications. Main available thermoplastics for microfluidics are suggested as material choices for device fabrication. The aim of this review is to explore DM and RP technologies for fabrication of an OoC with an embedded membrane after the evaluation of the main limitations of PDMS soft-lithography strategy. Different material options are also reviewed, as well as various bonding strategies. Finally, a new functional OoC device is showed, defining protocols for its fabrication in Cyclic Olefin Polymer (COP) using two different RP technologies. Different cells are seeded in both sides of the membrane as a proof of concept to test the optical and fluidic properties of the device.

## 1. Introduction

During the last two decades, the vast majority of microfluidic systems were built by soft lithography, a technique based on PolyDiMethylSiloxane (PDMS) micro-molding [1]. PDMS features some key properties that have made it to become the material of choice in microfluidics labs. To name some of them: (1) biocompatible; (2) optical clearance; (3) gas-permeability; and (4) patternable at high resolution [1]. 

Nevertheless, in the last few years, a few limitations have emerged, raising concerns about PDMS as an appropriate material for future production of Organ-on-a-Chip (OoC) devices, particularly, regarding its suitability for cell culture and the need of an easy manufacturability for both research and commercialization.

One of the main concerns is related to its bulk absorption of small molecules compared to the surface-only adsorption of glass and thermoplastics. PDMS was reported to absorb and deplete both estrogens [2] and administered drugs [3] in the media, altering their effectiveness. Thus, it can affect not only fundamental biology studies, but also drug discovery and high-throughput screening applications that rely on platform materials [4].

Another important point is leaching. PDMS in cured form contains residual uncross-linked polymer chains that can freely diffuse within the bulk material and can leach out into solution. Leached oligomers have been reported to incorporate into cell membranes [2]. The issue has received small attention to date; however, it is expected to become more evident as microfluidic systems become more popular for studies of cell membranes [4] in OoC applications.

Another challenge to consider when working with PDMS is the evaporation. It is inherently present in regular macroscale cell cultures, but the phenomenon becomes more dominant at the microscale where small amounts of liquid evaporation can significantly shift volumes, concentrations, chemical balances, and critical gradients among other factors [4,5,6,7]. Furthermore, this loss of volume can lead to bubbles that can propagate along with the system, block the flow, or lyse cells [4].

The PDMS oxygen permeability is three orders of magnitude greater than polystyrene of standard Petri dishes and this renders PDMS microchannels better suited for cell culture. However, considering the oxygen diffusion rates, PDMS-based chips may produce a hyperoxic microenvironment, causing cell stress [8,9]. 

In addition, PDMS is not considered the best candidate for a large-scale manufacturing regarding the fabrication process because it requires the molds that can be very expensive if they are fabricated using cleanroom facilities, and as a consequence, requires a significant amount of time from the design to the curing of the final device. The fabrication technique can be crucial also in academic research since not all the groups that work with OoC devices have access to cleanroom services as a matter of expertise or cost. The possibility of buying standard microfluidic devices at a low price is also an option, but if we want direct control over the experiments, there is usually the necessity to build tailored chips, particularly in the bioengineering field.

As an alternative, the use of thermoplastics may overcome some drawbacks mentioned before, still conserving some of the PDMS properties such as optical clearance, biocompatibility, and chemical stability. By all of thermoplastics, PolyStyrene (PS), Cyclic Olefin Polymer (COP), and Cyclic Olefin Copolymer (COC) may be the more desirable materials for microfluidic devices because they do not absorb hydrophobic drugs [3,10,11,12,13]. Mild plasma treatment on PS and COP (final contact angle of 19–36°) helps to achieve good cell growth, similar to commercial ones [14]. While PS has become a standard for cell culture, it was found to exert biological effects on the samples [15,16,17]. On the contrary, COP was reported as biologically inert [18,19,20]. Its ultra-low water vapor permeability (0.01% in 24 h), also relatively low compared to other gases such as oxygen [17,21], favors cell culture while limiting sample evaporation. Moreover, COP is highly transparent in the visible and near-ultraviolet regions of the spectrum [18] and has low autofluorescence [22]. 

The use of this material also includes the possibility to process the device fabrication in a more straightforward and scalable manner, using Rapid-Prototyping (RP) techniques and rendering cost-effective devices [23]. Hard plastics open a wide variety of manufacturing procedures based on Digital Manufacturing (DM). These processes are based on customer demand thanks to the support of digital technologies [24]. Sketches can be digitally inspected, adjusted, annotated, and cloud-shared with collaborators, resulting in a better design, cost, and performable [25] approach. All DM methods share (a) the ability to produce a physical device from a digital design file and (b) the ability to encode the fabrication process as a set of parameters, and, importantly, (c) the design file and the process parameters can be electronically sent to distant collaborators operating a similar machine in order to produce a replica of the same device [25]. A similar shift is desirable in microfluidics and OoC, where standardization and digitalization of fabrication processes could benefit design workflow for different applications. 

These techniques are usually divided into additive or subtractive manufacturing, depending on if the final part is obtained by joining material together, usually layer by layer, or withdrawing it from a bulk block. Subtractive manufacturing was implemented in microfluidics since the very beginning when the first devices were produced using methods derived from microsystem technologies such as silicon or glass etching [25,26,27]. There are subtractive techniques, like Xurography or laser-cut, by which, although used as part of an additive process, the final part is obtained by assembling different patterned layers. This process is often referred as Laminated Object Manufacturing (LOM) or sheet lamination. Employing Xurography for the fabrication of microfluidics devices, features down to 6 μm width and 25 μm thick can be obtained in minutes with no masks [28]. CO_2_ laser-cut is used for processing different materials, like PMMA (Poly-Methyl-MethAcrylate) [29], PDMS [30], Mylar [31,32,33,34,35,36], and Poly-lactic acid (PLA) [37,38]. Among the techniques used to bond two different layers, we can find heat press [38], heat lamination [28], Pressure-Sensitive Adhesive (PSA) [39,40], or solvent bonding [41].

Therefore, in this paper, we propose some strategies for an easy implementation, cost-efficacy, and fast realization (~15 min) of multi-layered OoC devices minimizing the uses of cleanroom facilities and high-end equipment. Some thermoplastics alternatives to PDMS, such as PC (Poly-Carbonate), PS, PMMA, and Zeonor^®^ 1420R COP, have been explored together with pattern fabrication techniques based on both laser-cut and Xurography. Finally, we have evaluated different layer bonding strategies. Results on cell seeding and culture confirm the appropriateness for the selected technology for OoC applications.

## 2. Materials and Methods

### 2.1. Materials

COP sheets (Zeonor^®^ 1420R, thickness 180 μm) are from Zeon Corporation (Tokyo, Japan). PC and PS (thickness 1 mm) were from Goodfellow (Huntingdon, England). PMMA (clear, thickness 3 mm) was from Trotec Laser Inc. (Marchtrenk, Austria). Cyclohexane (28920), Trichloromethane (02487), (3-Aminopropyl)triethoxysilane (APTES, ≥98%, A3648), and PC membranes (Whatman 9100–4710, pore size 1 μm, thickness 10 μm) were from Sigma Aldrich (St. Louis, MO, USA). Double-sided biocompatible Pressure-Sensitive Adhesive ARSeal^®^ 8026 was from Adhesive Research (Glen Rock, PA, USA). Adhesive transfer tape 467 MP was from 3M™ (Maplewood, MN, USA). Human BBB hCMEC/D3 endothelial cell line (SCC066) was from Merck Millipore (Burlington, MA, USA). 

Bovine pericytes were kindly donated by Ernest Giralt. The following products were obtained from Sigma Aldrich U.S.: collagen type-I solution from rat tail (#C3867), gelatin from porcine skin (#G1890), paraformaldehyde (PFA, #P6148), sodium azide (#71290), and Triton (#T8787). Phosphate buffer saline (PBS, Gibco^®^ #21600-010) and fetal bovine serum (FBS, #26140079) were obtained from Gibco^®^, Thermo Fisher Scientific, U.S. Antibodies were used from the following providers: anti-VE-cadherin antibody (Abcam #ab33168, UK), Alexa Fluor 568 goat anti-rabbit (Invitrogen™ #A-11036, Waltham, MA, USA), Alexa Fluor 488 ZO-1 monoclonal antibody (Invitrogen™ #339188, Waltham, MA, USA), Hoechst 33342 (Invitrogen™ #H3570, Waltham, MA, USA).

### 2.2. Methods

#### 2.2.1. Direct Polymer Bonding and APTES Functionalization

O_2_ plasma bonding, UV Ozone (UVO_3_), solvent bonding, and adhesive were explored as bonding strategies. COP, PC, PS, PMMA were tested as bulk material. In addition to them, a bonding test against PC membranes was also performed. APTES-functionalized PC membranes were used for plasma and UVO_3_. It consisted of modifying the surface of the material with a silane composite which could be later oxidized when exposing the hydroxyl (OH) groups. APTES functionalization and plasma activation bonding were reported by Aran et al. [42] as a method for embedding porous polymer membranes to a microfluidic device. This chemical was used as a crosslinker, showing a strong and irreversible bond between the membranes and microfluidic structure, with no significant alteration of membrane transport function or pore morphology.

O_2_ plasma processing was performed, using a Harrick Plasma Cleaner PDC-002-CE (Harrick Plasma, Ithaca, NY, USA) with O_2_ gas. Samples were treated with O_2_ plasma (t=[1,2,5] min, 29.6 W,0.2 Torr) after cleaning, brought into contact, and left at 65 °C for at least 10 min. UVO_3_ treatment was performed using UV/Ozone Cleaner (BioForce Nanoscience, Ames, IA, USA, t=[2,10,20] min). After treatment, samples were brought into contact and left at 65 °C for at least 15 min. 

Cyclohexane for COP and Trichloromethane for all other materials were used for solvent bonding. Different material combinations were tested: (1) COP on COP, (2) COP on PC membrane, (3) PC on PC membrane, (4) PMMA on PC, (5) PMMA on PMMA, (6) PS on PS, (7) PS on PC membrane. Substrates were suspended 2 3 mm above a glass Petri dish filled with the solvent. Another glass Petri dish was flipped on top to cover the set-up. After exposing to vapors at 65 °C, the substrates were pressed together and left at 65 °C.

APTES-functionalized PC membranes were prepared following similar protocol as described by Aran et al. [42]. Briefly, a solution of 5% APTES in DeIonized (DI) water was pre-heated in a glass Petri dish at 80 °C and covered with another Petri dish to prevent evaporation. The membrane was activated using O_2_ plasma (1 min,29.6 W,0.2 Torr) and immediately submersed in the solution. After 20 min, the membrane was washed thoroughly using DI water, deposited on top of a PTFE (Poly-Tetra-Fluoro-Ethylene) flat substrate, and dried using N_2_.

#### 2.2.2. COP Contact Angle Measurement

Measurements were performed using OCA15 Pro system from DataPhysics (Filderstadt, Germany). After focusing the sample, 2-μL drop of de-ionized water was released on top of the surface for digital image capture. Contact angle measurements were performed using SCA 20 software from DataPhysics.

#### 2.2.3. OoC Design and Fabrication

**Figure 1:**
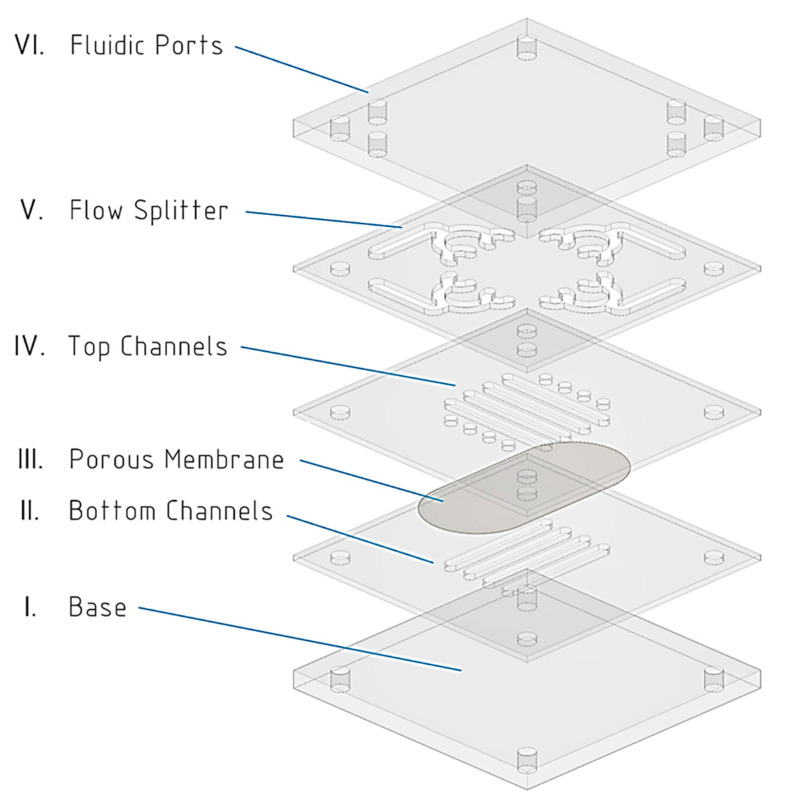
Cyclic Olefin Polymer (COP) device exploded view. From bottom to top: (I) base layer (COP); (II) lower channels layer (COP); (III) Poly-Carbonate (PC) porous membrane; (IV) upper channels layer (COP); (V) flow splitters (COP); (VI) inlets/outlets layer (COP/Poly-Methyl-MethAcrylate (PMMA)).

**Figure A1:**
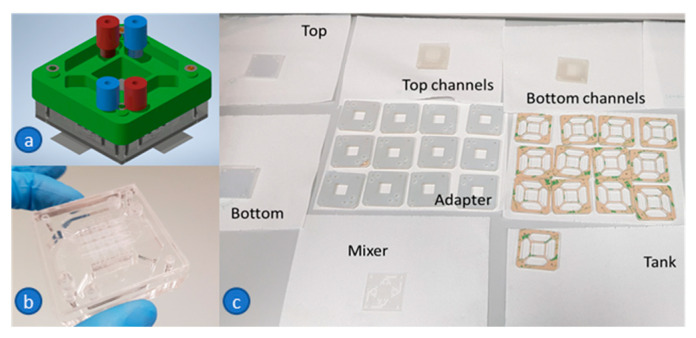
(**a**) Autodesk Inventor^®^ design of the COP device with connectors; (**b**) Final assembled device; (**c**) Laser-cut parts for a batch of devices.

We designed a multilayered device using Autodesk Inventor^®^. The device consists of a PC membrane sandwiched between two COP layers, each containing network of 4 parallel microfluidic channels (Figure 1 and Figure A1). Flow is distributed through these parallel channels (layers II and IV) thanks to a fluidic splitter on layer (V). Cross-section dimensions for individual channels are 2 mm×180 μm, length is 20 mm. Design includes Mini Luer fluidic ports in a 3-mm PMMA layer (VII), to facilitate OoC couple to standard connectors.

After removing the protective liner of a 180-μm thick COP sheet, the substrate was rinsed with IPA (2-propanol) and dried using N_2_. Double-sided PSA was laminated over the COP and PMMA surfaces with the help of a hand-roller, avoiding bubble formations.

Layers were customized using Graphtec^®^ CE-5000-40 vinyl cutter (Graphtec Corporation, Tokyo, Japan). Cutting was performed using 2 passes at a speed of $30\;{\mathrm{mms}}^{-1}\;$with a mechanical precision of 5 microns, allowing a lateral resolution of 25 microns.

A Trotec Speedy 100 laser cutter (Trotec Laser Inc., Marchtrenk, Austria) was used for laser-cut fabrication. Layers were cut using 2-mm lens (40 W,5000 Hz, speed: $0.15{\;\mathrm{ms}}^{-1}$). Thick layers of PMMA were cut with adhesive side facing up. ¼-28 UNF threads were formed using a threading plug tap.

Assembly was performed in a custom-made aluminum aligner. It consisted of a thick base with 4 press-fitted steel dowel pins and a thick cover with 4 holes (both 60×60×10 mm). Layers located below the membrane were placed in the aligner base (Figure A1). Aligner cover was placed on top and the whole stack was pressed using an Atlas T8 hydraulic press (Specac Ltd., Orpington, UK) at $2\times 10^{3}\;\mathrm{kg}\;$for 30 s. This procedure was repeated for the layers located above the membrane. Finally, a thicker PMMA layer with mechanized Mini-Luer^®^ ports was assembled to obtain the complete device.

#### 2.2.4. Cell Culture for OoC Application

Each device was activated using the Bioforce UVO_3_ tip cleaner (20 min) under a fume hood. The device was connected to a 10 mL disposable syringe (BBraun, Rubi, Spain) using Luer 23 G flat needles (Instechlabs, Leipzig-Markkleeberg, Germany), Tygon^®^ tubing (VWR International Eurolab, Barcelona, Spain), and ¼-28 UNF fluidic connectors (microfluidic ChipShop, Jena, Germany). Inlet reservoirs were filled sequentially with Ethanol (EtOH) 70% and PBS solution to rinse the device microchannels by negative pressure-induced flow. Then, the device was placed onto a hotplate in order to maintain the temperature at 37 °C.

Subsequently, the microdevices were functionalized. The inlet reservoir was filled with 500 μL of coating solution, connecting the pump in refill mode (volume: 200 μL, flowrate: $-200{\;\mu L\;\min}^{-1}$). Lower channels were functionalized, incubating them with 1:20 collagen solution in PBS (1 h,37 °C), and upper channels were functionalized with a $2\;\mathrm{mg}\cdot {\mathrm{mL}}^{-1}$ gelatin solution in PBS (15 min,37 °C). Afterwards, cell suspension of hCMEC/D3 cells in endothelial cells culture medium was inserted at the bottom channel ($2.8\times 10^{6}\;\mathrm{cells}\cdot {\mathrm{mL}}^{-1}$) and the devices were left incubating in a humidified CO_2_ incubator at 37 °C for at least 2 h, flipped upside-down to promote cell adhesion onto the membrane. Then, a cell suspension of pericytes in pericyte culture medium was inserted at the upper channel ($2\times 10^{6\;}\mathrm{cells}\cdot {\mathrm{mL}}^{-1}$). The devices were left incubating in the incubator at 37 °C for at least 2 h before filling each reservoir with the corresponding medium and covering them with a lid.

The devices were left in a humidified incubator at (37 °C, $5{\%\;\mathrm{CO}}_{2}$) with no flow and new media was perfused 50 µL/min every 24 h through negative pressure-driven flow. After 5 days in vitro, the devices were fixed in a 4% PFA solution for 30 min at room temperature, then rinsed in PBS and kept in PBS with 0.02% of sodium azide.

To perform the immunostaining, all solutions were perfused with a negative pressure-driven flow of 50 µL/min. Cells were permeabilized with a 0.1% Triton in PBS solution. After incubating with blocking solution—PBS with 0.1% Triton and 10% FBS—for 2 h, cells were washed and incubated with primary antibody (rabbit polyclonal anti-VE-cadherin antibody, 1:1000) overnight at 4 °C. Then cells were washed and incubated with secondary antibody (Alexa Fluor 568 goat anti-rabbit, 1:1000) for 1 h at room temperature protected from light, washed again, and incubated with the primary monoclonal antibody (Alexa Fluor 488 ZO-1 monoclonal antibody, 1:100) for 3 h at room temperature. After washing, samples were stained with 32.4 µM of Hoechst 33,342 for 10 min, washed, and kept in PBS-azide for imaging in an inverted confocal Leica SP5 microscope. Afterward, images were processed with ImageJ software.

## 3. Results

### 3.1. Direct Polymer Bonding

Effective bonding between various layers of the device is fundamental to avoid liquid leakages, which would compromise the entire device functionality. We tested COP bonding to different materials using Plasma treatment, UVO_3_ treatment, and solvent bonding. Results are summarized in Table 1. An extended study evaluating other thermoplastics bonding is shown in Table A1 of Appendix A.

O_2_ plasma and UVO_3_ gave generally poor bonding or no-bonding results. Using O_2_ plasma to seal two samples of the same bulk polymer failed with all materials. UVO_3_ treatment gave almost the same result, with exception of COP-COP bonding. Good strength COP-COP bonding was obtained with higher UVO_3_ exposure times and thermal post-curing. Both surface activation methods failed to bond PC membranes to bulk materials. Good results were obtained between COP and APTES-functionalized PC membranes. Sandwiching between two COP layers was not possible, as one of the layers always showed poor bonding.

Solvent bonding gave stronger results between same-material combinations (COP, PS, PMMA).

### 3.2. COP Contact Angle Measurements

**Figure 2:**
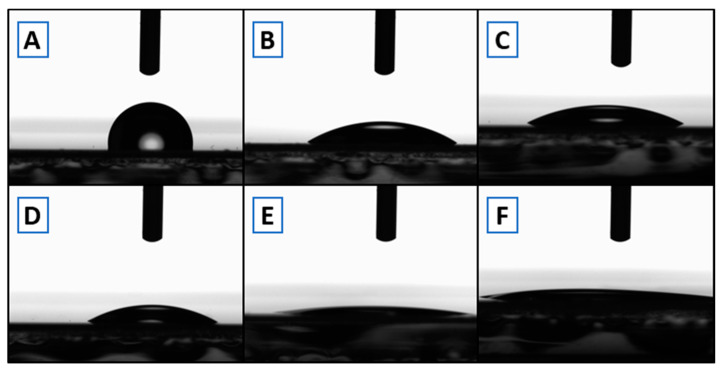
Contact angle measurement of COP. (**A**) Native; (**B**,**C**) after UVO_3_ surface treatment (10 and 20 min, respectively); (**D**–**F**) after O_2_ plasma treatment (1, 5, and 10 min, respectively).

Contact angle measurements (Figure 2) were performed on native COP after O_2_ plasma or UVO_3_ treatment to evaluate treatment effects on the material. Table 2 presents how the contact angle is significantly reduced after treatment in both cases, turning into a more hydrophilic COP surface.

### 3.3. Design and Fabrication

**Figure 3:**
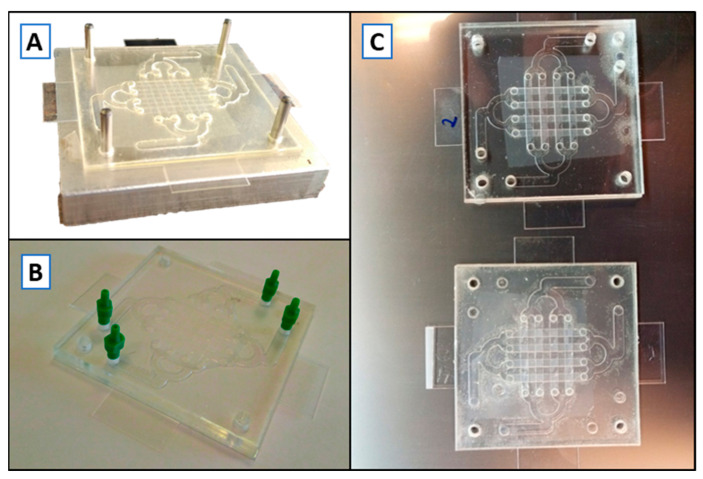
Organ-on-a-Chip (OoC) device assembly. (**A**) OoC in the alignment block during the assembly process; (**B**) Final OoC device assembled with green Mini-Luer^®^ connectors; (**C**) Optical clarity comparison between ARSeal 8026 PSA (top) and 467 MP (bottom).

We selected COP to fabricate our multilayered device using Xurography and laser-cut to pattern them. After the poor results obtained with the different solvent bonding approaches, adhesive bonding through 467 MP and adhesive layers were evaluated. Assembly was successful in both cases, though optical quality was better with ARSeal^®^ 8026, (Figure 3). Final assembled devices were successfully tested against leakage for 24 h. They were placed under an optical microscope and PBS/water plus colorant was pumped through the microchannels at 2 mL min^−1^.

### 3.4. Sensing Integration: O_2_ Concentration Sensing

**Figure A2:**
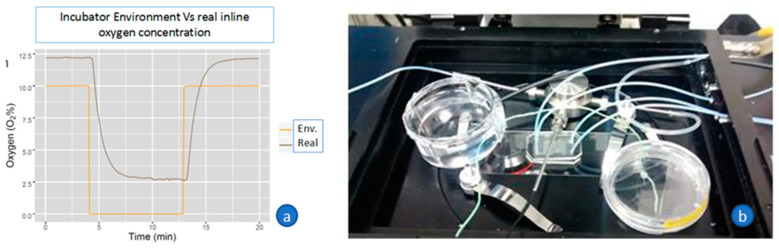
Oxygen monitoring. (**a**) System response; (**b**) Measuring setup.

PreSens^®^ flow-through cells were enclosed in the setup, before and after the chip outlets, to measure the oxygen input and output to/from the device through the tubing. Input concentration was controlled by the microscope incubator chamber and a silicone tubing coil used as gas and temperature interchanger. Response of oxygen concentration vs. incubator environment was characterized, as well as flowrate dependence of oxygen concentration (Figure A2).

### 3.5. Cell Culture for OoC Application 

**Figure 4:**
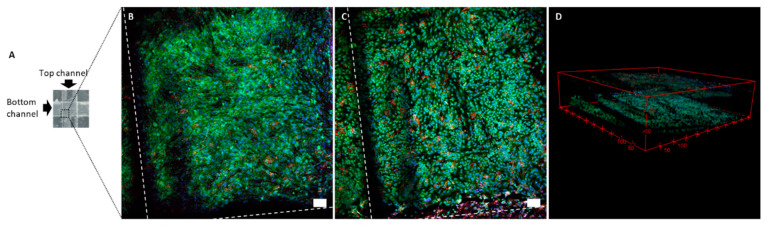
Confocal images of a cross-section of top and bottom channels. The diagram (**A**) indicates the area of a cross-section where the image was taken. Z-projection of the membrane position with endothelial cells and pericytes on opposite sides of the membrane (**B**), and bottom position with endothelial cells (**C**). 3D view of the cross-section (**D**). ZO-1 tight junctions are stained in green. VE-cadherin adherens junctions are stained in red. Nuclei are stained in blue. Scale bars are 100 µm. The white dashed line in confocal images indicates the shadow coming from the wall of the channels.

**Figure A3:**
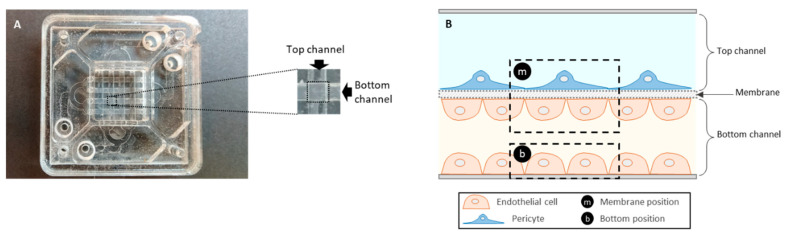
Schematic of cell-seeding areas in OoC device. (**A**) Top view of a μBBB device with an augmented image of the grid indicating an example of a top channel (vertical), bottom channel (horizontal), and a cross-section (indicated with a square in the centre). (**B**) Schematic of the transversal structure of each cross-section in the device. Pericytes are seeded in the top channel in contact with the polycarbonate membrane. Endothelial cells are seeded in the top channel, both in contact with the membrane and in contact with the bottom of the device. Each channel is supplied with its own cell-specific culture medium. The positions analysed for imaging purposes are indicated as bottom-position and membrane-position.

The microfluidic prototype can be used to mimic biological barriers and to analyze its permeability. Brain endothelial cells and pericytes were cultured on opposed sides of the device membrane, mimicking a simplified blood-brain barrier (Figure A3). The culture of both cell types was successful. After five days in vitro, the bottom channel was covered by an endothelial cell monolayer, and the top channel by pericytes as shown in confocal images of Figure 4. The staining of tight junctions, nuclei, and adherens junctions was performed from the bottom of the device using a confocal microscope, as a routine test for the blood-brain barrier. The devices showed excellent optical characteristics.

**Figure 5:**
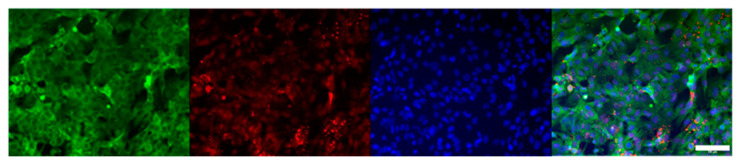
Immunostaining of endothelial cells cultured on a Petri dish. ZO-1 tight junctions are stained in green. VE-cadherin adherens junctions are stained in red. Nuclei are stained in blue. Scale bar 100 µm.

We have compared the results obtained in our device with the classical Petri dish culture (Figure 5), observing the excellent biocompatibility of our prototype. 

## 4. Discussion

The work presented here explores digital manufacturing technologies for OoC device fabrication to enable low-cost chip production for research purposes. Particularly, our focus was on LOM techniques, using Xurography and a CO_2_ laser-cut. After identifying materials following their biocompatible characteristics, we tested possible bonding strategies. It was not possible to achieve irreversible and solid bonding using O_2_ plasma on any pristine material, probably due to the relatively low energy of equipment. Although PMMA bonding with UVO_3_ and O_2_ plasma was previously reported [43], likely higher plasma power and pressures (0.2 1 Pa) were used. Plasma bonding of PC membranes to COP was successfully achieved only after APTES treatment. UVO_3_ and solvent bonding results on COP-COP were better, but the optical quality was low. Consequently, double-sided Pressure-Sensitive Adhesive is used as the bonding method between layers.

COP is the selected material for device layers fabrication due to its top-grade cell culture properties after mild plasma treatment (contact angle in 19 36° range) [14], discarding PC considering its flammability characteristics when exposed to CO_2_ laser processing and PS or PMMA for their poor optical properties. Contact angle measurements confirmed that similar surface activation was achieved after 1-min O_2_ plasma or 10–20 min UVO_3_ treatment.

Only a few works can be found in the microfluidics literature regarding processing COP with cutting plotter [41,44] or laser cutters [45], and even a Lab-on-Chip (LoC) device fabricated in COP using cutting plotter and PSA with an embedded white polyester membrane [46]. COP has a relatively low permeability to water vapor compared to other gases such as oxygen, limiting sample evaporation and favoring the culture of respiring cells [42,44]. COP as well as COC also show other benefits for microfluidics applications, like its high transparency in the visible and near-ultraviolet regions of the spectrum [43], low autofluorescence [45], enabling long-term stable surface treatments, and the deposition of metals by sputtering directly on the substrate.

We showed how both LOM fabrication strategies described here bring the benefits of RP into microfluidics fabrication. Throughput is improved by the reduction of process timing as well as reducing the number of intermediate steps. We reduced the overall process cost and especially costs related to future modifications of the device. We find adhesive bonding to be a versatile solution for rapid prototyping in the research environment, as it enables to extend the process to different membrane materials. On the contrary, solvent or surface activation strategies might limit the process to a smaller range of compatible materials or might require adding chemical functionalization to achieve bonding of different materials. Finally, the OoC technology developed was tested in biological applications to reproduce the blood-brain barrier in vitro by means of co-culture of two different types of cells.

## 5. Conclusions

Rapid prototyping methods showed here overcome most of the limitations of PDMS soft lithography. Thanks to the material of choice, COP, and the used methodology, the produced device shows excellent optical characteristics, and low autofluorescence. This fabrication process is rapid (~15 min), easier, low-cost, and enables modifications of the design, including scalability. On the other hand, the process allows an easy solution for embedding commercial established semi-permeable membranes to interconnect different channels or chambers. The technological process is compatible with membrane functionalization and cell culture, allowing to carry out Organ-on-a-Chip devices in an easier manner. 

## Figures and Tables

**Table 1:** Direct polymer bonding results for COP. (*) low optical quality; (**) Good results only on one side of the membrane, unable to obtain a proper polymer-PC membrane-polymer sandwich; (-) not tested.

1st Material				COP	
2nd Material			COP	PC	APTES—PC
	Low Power O_2_ Plasma	1 min	None	None	None
		2 min	None	None	Good **
		10 min	None	Poor	Good **
Method		5 min	None	None	None
	UVO_3_	10 min	Poor	None	Poor
		20 min	Good	None	Poor
	Solvent		Good *	None	-

**Table 2:** Contact angle measurements of COP, natively and after surface treatment. (Mean values and standard deviations for N = 3 samples).

Treatment	Native	UVO_3_		O_2_ Plasma		
Time [min]	-	10	20	1	5	10
C. A. [deg]	98.4 ± 1.53	32.83 ±1.18	24.41 ± 0.99	28.6 ± 2.23	8.03 ± 0.78	9.13 ± 1.31

## Appendix A

**Table A1:** Direct polymer bonding results. (*) low optical quality; (**) Good results only on one side of the membrane, unable to obtain a proper polymer-PC membrane-polymer sandwich; (-) not tested.

1st Material				COP			PS		PMMA		PC
2nd Material			COP	PC	APTES—PC	PS	PC	APTES—PC	PMMA	PC	PC
	Low Power O_2_ Plasma	1 min	None	None	None	-	-	-	None	None	None
		2 min	None	None	Good **	None	-	None	None	None	None
		10 min	None	Poor	Good **	None	None	None	None	None	None
Method		5 min	None	None	None	None	None	None	None	None	None
	UVO_3_	10 min	Poor	None	Poor	None	-	-	None	None	None
		20 min	Good	None	Poor	None	None	None	None	None	None
	Solvent		Good *	None	-	Good *	None	-	Good *	None	Poor
